# Genome-resolved metagenomics reveals novel archaeal and bacterial genomes from Amazonian forest and pasture soils

**DOI:** 10.1099/mgen.0.000853

**Published:** 2022-07-27

**Authors:** Andressa M. Venturini, Júlia B. Gontijo, Jéssica A. Mandro, Fabiana S. Paula, Caio A. Yoshiura, Aline G. da França, Siu M. Tsai

**Affiliations:** ^1^​ Cell and Molecular Biology Laboratory, Center for Nuclear Energy in Agriculture, University of São Paulo, Piracicaba, SP, Brazil; ^2^​ Princeton Institute for International and Regional Studies, Princeton University, Princeton, NJ, USA; ^3^​ Department of Biological Oceanography, Oceanographic Institute, University of São Paulo, São Paulo, SP, Brazil

**Keywords:** forest-to-pasture conversion, land-use change, metagenome-assembled genomes, methane cycle, nitrogen cycle, soil microbial communities

## Abstract

Amazonian soil microbial communities are known to be affected by the forest-to-pasture conversion, but the identity and metabolic potential of most of their organisms remain poorly characterized. To contribute to the understanding of these communities, here we describe metagenome-assembled genomes (MAGs) recovered from 12 forest and pasture soil metagenomes of the Brazilian Eastern Amazon. We obtained 11 forest and 30 pasture MAGs (≥50% of completeness and ≤10 % of contamination), distributed among two archaeal and 11 bacterial phyla. The taxonomic classification results suggest that most MAGs may represent potential novel microbial taxa. MAGs selected for further evaluation included members of *Acidobacteriota *, *Actinobacteriota *, *Desulfobacterota_B*, *Desulfobacterota_F*, *Dormibacterota*, *Eremiobacterota *, *Halobacteriota*, *Proteobacteria *, and *Thermoproteota *, thus revealing their roles in carbohydrate degradation and mercury detoxification as well as in the sulphur, nitrogen, and methane cycles. A methane-producing *Archaea* of the genus *Methanosarcina* was almost exclusively recovered from pasture soils, which can be linked to a sink-to-source shift after the forest-to-pasture conversion. The novel MAGs constitute an important resource to help us unravel the yet-unknown microbial diversity in Amazonian soils and its functional potential and, consequently, the responses of these microorganisms to land-use change.

## Data Summary

The metadata, metagenomic reads, and the sequences of the metagenome-assembled genomes are available on the KBase platform at https://www.doi.org/10.25982/91640.61/1870603 and on NCBI under the umbrella project PRJNA842732 at https://www.ncbi.nlm.nih.gov/bioproject/PRJNA842732. The metadata and the raw forward metagenomic reads can also be found under the project “Metagenomic sequencing of forest and pasture soils of Eastern Amazon under different treatments - Forward reads” (mgp83208) in the MG-Rast server 4.0.3. at https://www.mg-rast.org. Other supporting data are available in the Supplementary Material of this article.

**Impact Statement**

The soil microbial communities of the Amazon rainforest have been evaluated in the context of deforestation and land-use change, but their diversity remains largely unknown. In this paper, 41 metagenome-assembled genomes (MAGs) (≥50 % of completeness and ≤10 % of contamination) were recovered from forest and pasture soils and characterized. The MAGs were spread over 11 bacterial and two archaeal phyla, 90% and 29 % of which could not be assigned to any known species and genus, respectively. Gene annotations indicated their potential roles in biogeochemical cycling, mercury detoxification, and the degradation of complex carbohydrates, revealing distinct functional patterns between forest and pasture soil microbial communities.

## Introduction

Soil microorganisms play crucial roles in below- and above-ground ecosystems, including the formation and stabilization of soil aggregates, carbon storage, organic matter decomposition, nutrient cycling, soil fertility, plant growth and health [1, 2], and even the production and consumption of greenhouse gases, such as methane (CH_4_) and nitrous oxide (N_2_O) [3, 4]. Soil microbes are also considered important components of soil health and have been used as bioindicators in soil-health evaluations [2, 5]. However, despite their ecological and economic impacts, assessments on biodiversity have neglected soil macro- and microorganisms [6]. From both diversity and functional perspectives, soils from tropical and subtropical regions are even less studied [6, 7], including the Amazon rainforest.

The Amazon is one of the most important reservoirs of biodiversity on Earth [8]. Due to the increase in deforestation in recent years [9] and the international concern regarding the future of this rainforest, several studies have been carried out to examine the effects of forest clearing and conversion on its soil physical, chemical, and biological attributes. These studies have revealed that the forest-to-pasture conversion alters the abundance, taxonomic and functional profiles of soil microbial communities [e.g. 10, 11, 12], therefore impacting several environmental processes, such as the soil CH_4_ cycling and fluxes [e.g. 13, 14, 15]. Nevertheless, to date, the identity and metabolic potential of a considerable fraction of the Amazonian soil microbial communities remain unknown, which limits our understanding of land-use impacts on these organisms and the biogeochemical cycles they drive.

In this context, genome-resolved metagenomics can be used to assemble overlapping short reads into longer contiguous sequences (contigs) and group them (binning) into putative metagenome-assembled genomes (MAGs) [16]. MAGs can then be taxonomically classified and functionally annotated using curated databases, helping us identify and understand the potential roles of yet-to-be cultivable microorganisms in the environment. This approach has expanded the known microbial phylogenetic diversity [e.g. 17, 18, 19], thus rapidly transforming the field of microbiology. Nevertheless, only a small number of studies have used this method so far to recover archaeal and bacterial MAGs from Amazonian soils [20, 21].

In this study, we used genome-resolved metagenomics to assemble and recover archaeal and bacterial MAGs from forest and pasture soils of the Brazilian Eastern Amazon. This approach was carried out using shotgun metagenomic sequencing data from a microcosm experiment in which soil moisture levels were increased. This experiment was previously conducted to evaluate the combined effects of forest-to-pasture conversion and increased moisture on soil CH_4_ microbial communities [15]. The genomes described here provide an important resource for the characterization of the microbial communities in Amazonian soils and, consequently, for our understanding of their responses to land-use changes and other environmental disturbances.

## Methods

### Site description, soil sampling, and microcosm experiment

The soil sampling was carried out in July 2015 in a pristine forest of the Tapajós National Forest (3°17'44.4"S 54°57'46.7"W) and an active cattle pasture (3°18'46.7"S 54°54'34.8"W), in the state of Pará, in the Brazilian Eastern Amazon. Following the removal of the litter layer, soil samples from 0 to 10 cm depth were collected in three sampling points per site, each separated by 50 m. Samples from each land-use treatment were combined, sieved through a 5 mm mesh sieve, and subjected to a microcosm experiment under increasing soil moisture levels. The microcosms were maintained and monitored for 30 days at 25 °C in a Biochemical Oxygen Demand incubator. Moisture treatments were established in triplicate for each land use using 1.5 litre glass jars filled with 350 g of soil each. These treatments included the original soil gravimetric moisture of each site (22 % for forest and 24 % for pasture) and 100 % of gravimetric moisture at field capacity (50 % for forest and 63 % for pasture). Soil samples from each microcosm were frozen in liquid nitrogen at the end of the experiment and stored at −80 °C.

### DNA extraction, quantification, and sequencing

Forest and pasture soil samples under original soil moisture and at 100 % field capacity were DNA-extracted in duplicate using the PowerLyzer PowerSoil DNA Isolation Kit (QIAGEN, Hilden, North Rhine-Westphalia, Germany), totaling 12 DNA samples, following a protocol optimized for Amazon soils [22]. DNA samples were checked using 1 % agarose gel electrophoresis and a Nanodrop 2000c spectrophotometer (Thermo Fisher Scientific, Inc., Waltham, MA, USA) and stored at −20 °C. Paired-end shotgun metagenomic sequencing (2×150 bp) was performed on an Illumina HiSeq platform (Illumina, Inc., San Diego, CA, USA) at Novogene Co., Ltd. (Beijing, China), using the NEBNext Ultra II DNA Library Prep Kit for Illumina (New England Biolabs, Inc., Ipswich, MA, USA) for library construction. Detailed information about the sites, their soil physicochemical properties, the design of the microcosm experiment, and DNA extraction and sequencing was previously reported [15, 21].

### Recovery and characterization of MAGs

The bioinformatics analysis was performed on the KBase platform [23]. Metagenomic sequences were uploaded to the platform and imported into a narrative as paired-end reads using the KBase apps Upload File to Staging from Web v1.0.12 and Import FASTQ/SRA File as Reads from Staging Area [23], respectively. Paired-end reads were quality-checked with FastQC v0.11.5 [24] and, outside KBase [23], with MultiQC [25]. Based on the results, reads were cleaned from adaptors, trimmed, and filtered using Trimmomatic v0.36 (altered parameters: adapters, TruSeq3-PE-2; sliding window minimum quality, 20; head crop length, 10; minimum read length, 50) [26]. The remaining paired-end reads from each land use (regardless of the soil moisture treatment) were again quality-checked with FastQC [24] and MultiQC [25] and merged into one object using the KBase app Merge Reads Libraries v1.0.1 [23].

Bins from each land use were recovered through co-assembly using MEGAHIT v1.2.9 (altered parameter: preset, meta-large) [27] and binning using MetaBAT2 v1.7 [28], MaxBin2 v2.2.4 (altered parameters: marker set, both 107 and 40 marker genes; minimum contig length, 2500 bp; plot markers per contig) [29], and CONCOCT v1.1 [30]. The resulting bins of each land use were optimized using the app DAS Tool v1.1.2 [31]. DAS Tool bins were quality-checked (altered parameter: reference tree, full tree) and filtered (altered parameters: reference tree, full tree; completeness, ≥50 %; contamination, ≤10 %) using CheckM v1.0.18 apps to keep only bins with moderate/substantial/near completeness and medium/low contamination, as defined by Parks *et al*. [32]. Based on these scores, the quality of each bin (completeness − 5 × contamination) was calculated. The remaining bins were taxonomically classified using Classify Microbes with GTDB-Tk v1.7.0 (release R06-RS202) [33]. If necessary, bins were additionally compared against closely related genomes using the app Compute ANI with FastANI v.0.1.3 [34].

Bins with the values of completeness (>90 %) and contamination (<5 %) for high-quality drafts of the Minimum Information about a Metagenome-Assembled Genome (MIMAG) standards [35] were selected for further analysis and extracted as assemblies using the KBase app Extract Bins as Assemblies from BinnedContigs v1.0.2 [23]. Two pasture MAGs (Bin.006_Pasture of the family *Binataceae* and Bin.035_Pasture of the genus *Methanosarcina *) that did not meet these criteria were also selected due to the relevance of both groups in the CH_4_ cycle [36, 37], totaling 12 MAGs selected for additional exploration. The relative abundance of each selected bin in both merged forest and pasture metagenomes (defined as the number of mapped reads divided by the number of reads in the metagenome) was estimated using Bowtie2 v2.3.2 (altered parameter: alignment type preset options, very-sensitive) [38].

A set with all the selected bins was created using the KBase app Build AssemblySet v1.0.1 [23]. Bins were annotated by the beta app Annotate and Distill Assemblies with DRAM (Distilled and Refined Annotation of Metabolism) [39]. DRAM provides annotations of MAGs using multiple databases and then summarizes the results to facilitate the exploration of their functional and structural traits, also using functional marker genes to infer metabolic descriptors of MAGs [39]. More detailed information about the functioning of DRAM can be found here: https://github.com/WrightonLabCSU/DRAM/wiki/1.-How-DRAM-Works. The presence of 23S, 16S, and 5S ribosomal RNA (rRNA) genes and transfer RNAs (tRNAs) in each MAG was also checked by DRAM. Figures were generated using the packages ggplot2 3.3.5 [40] and ggalluvial 0.12.3 [41] in R 4.0.2 [42].

## Results and discussion

### Sequencing and co-assembly statistics

The shotgun metagenomic sequencing of the 12 DNA samples from forest and pasture soils resulted in 594.9 million paired-end reads of 150 bp in length, with an average of 49.6 million per sample, ranging from 39.9 to 79.9 million across samples. After our quality control, 529.9 million paired-end reads from 50 to 140 bp in length were kept, with an average of 44.2 million per sample, ranging from 34.8 to 72.9 million. After merging the reads per land use, the forest and pasture libraries had 273.3 and 256.6 million paired-end reads, respectively.

**Fig. 1.**
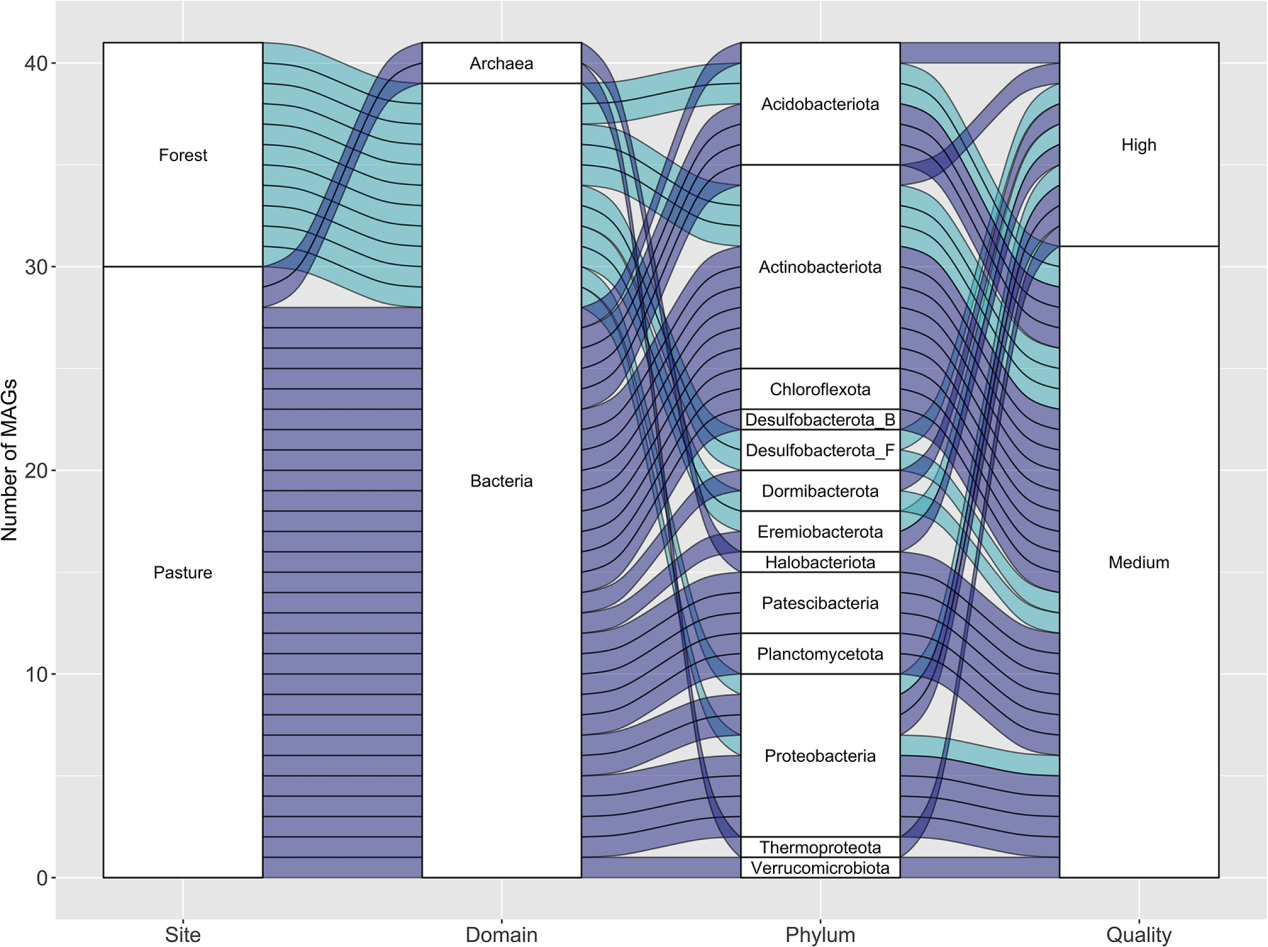
Taxonomic classification (at the domain and phylum levels) and quality (based on completeness and contamination scores) of the metagenome-assembled genomes (MAGs) from the merged forest and pasture metagenomes.

The co-assembly of the forest paired-end reads generated a total of 367 892 contigs (374 273 bp in the largest contig), with N50 of 3 480 bp and L50 of 106 965 bp, while for pasture, it produced 366 749 contigs (515 562 bp in the largest contig), with N50 of 4 207 bp and L50 of 89 012 bp. A total of 19 forest and 39 pasture bins resulted from DAS Tool [31], in which 11 and 30, respectively, passed our quality filter (≥50 % of completeness and ≤10 % of contamination) (Fig. 1 and Table S1, available in the online version of this article).

### Taxonomic assignment and relative abundance of MAGs

Regarding the taxonomic classification of the bins, 39 belonged to 11 bacterial phyla: *Actinobacteriota *, *Proteobacteria *, *Acidobacteriota *, *Patescibacteria *, *Chloroflexota *, *Desulfobacterota_F*, *Dormibacterota*, *Eremiobacterota *, *Planctomycetota *, *Desulfobacterota_B*, and *Verrucomicrobiota* (Fig. 1 and Table S1, available in the online version of this article). We also recovered archaeal bins from the phyla *Halobacteriota* and *Thermoproteota *. Only four bins could be classified at the species level. In fact, 10 bacterial bins could not be assigned at the genus level and two at the family level, thus demonstrating the potential of this approach to reveal the yet-unknown microbial diversity of Amazonian soils. It is important to mention that, using other bioinformatics tools, Lemos *et al*. [21] previously recovered and characterized two patescibacterial MAGs from our high-moisture pasture soils, and additional tests based on the average nucleotide identity (ANI) indicated that two of our genomes belong to those species (99.9 % for Bin.036_Pasture and WARW01 000 000 (used as reference and available at https://www.ncbi.nlm.nih.gov/nuccore/WARW00000000.1/), and 99.9 % for Bin.038_Pasture and WARV01000000 (used as reference and available at https://www.ncbi.nlm.nih.gov/nuccore/WARV00000000)).

**Fig. 2.**
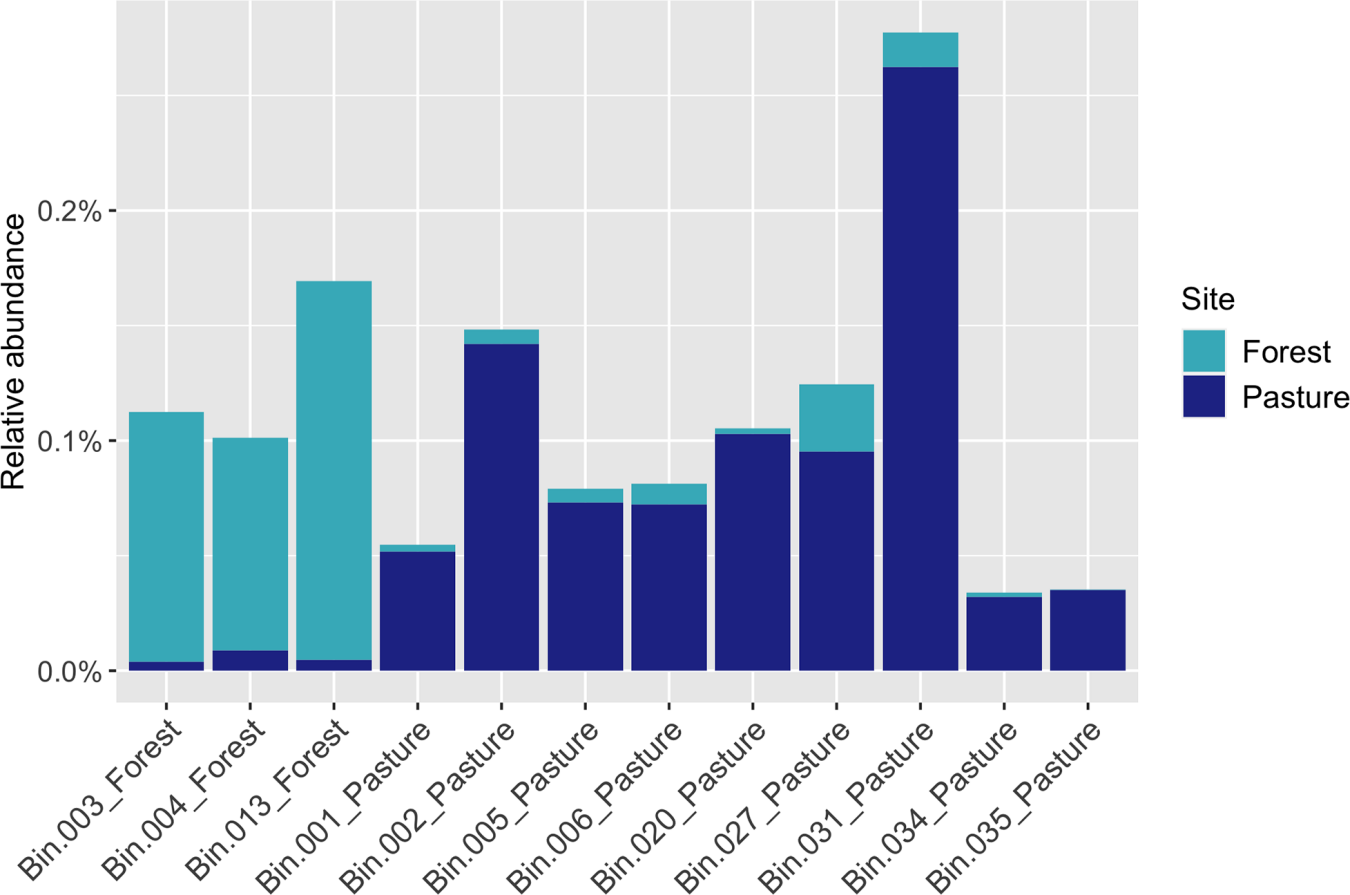
Relative abundance (%) of each selected metagenome-assembled genome (MAG) in the merged forest and pasture metagenomes. The MAGs were selected based on their completeness (>90 %) and contamination (<5 %) scores. Bin.006_Pasture and Bin.035_Pasture were also included due to their environmental relevance.

A total of 12 MAGs were selected and extracted as assemblies for further analysis due to their completeness (>90 %) and contamination (<5 %) scores or environmental importance: three MAGs from the forest and nine from the pasture (Table 1). Proteobacterial high-quality MAGs belonged to the classes *Alphaproteobacteria* (Bin.027_Pasture and Bin.031_Pasture) and *Gammaproteobacteria* (Bin.013_Forest). The classes *Acidimicrobiia* from *Actinobacteriota* (Bin.002_Pasture), *Acidobacteriae* from *Acidobacteriota* (Bin.001_Pasture), *Binatia* from *Desulfobacterota* _B (Bin.006_Pasture), *Desulfuromonadia* from *Desulfobacterota* _F (Bin.003_Forest), *Dormibacteria* from *Dormibacterota* (Bin.020_Pasture), *Eremiobacteria* from *Eremiobacterota* (Bin.004_Forest and Bin.005_Pasture), *Methanosarcinia* from *Halobacteriota* (Bin.035_Pasture), and *Nitrososphaeria* from *Thermoproteota* (Bin.034_Pasture) were also found. Bin.013_Forest had the highest relative abundance in our merged forest metagenome, and Bin.031_Pasture in the pasture metagenome (Table 1 and Fig. 2).

**Table 1.** Detailed information (taxonomic classification, completeness, contamination, GC content, genome size, number of contigs, relative abundance in relation to its merged metagenome of origin, number of 5S and 16S rRNA genes, number of tRNAs and tRNAs for different amino acids) of the selected forest and pasture metagenome-assembled genomes (MAGs).

MAG	Site	GTDB classification	Completeness (%)	Contamination (%)	GC content (%)	Genome size (bp)	No. of contigs	Relative abundance (%)	No. of 5S rRNA	No. of 16S rRNA	No. of tRNAs	No. of tRNAs for different amino acids
Bin.003_Forest	Forest	d_Bacteria p_Desulfobacterota_F c_Desulfuromonadia o_Geobacterales f_Geobacteraceae	95.7	1.9	59.0	4438015	103	0.11	2	0	50	18
Bin.004_Forest	Forest	d_Bacteria p_Eremiobacterota c_Eremiobacteria o_Baltobacterales f_Baltobacteraceae g_Aquilonibacter	93.8	3.5	59.3	3739922	442	0.09	1	0	39	18
Bin.013_Forest	Forest	d_Bacteria p_Proteobacteria c_Gammaproteobacteria o_Burkholderiales f_Burkholderiaceae g_Paraburkholderia s_Paraburkholderia sp004298475	95.7	3.8	63.5	5591535	78	0.16	0	0	48	18
Bin.001_Pasture	Pasture	d_Bacteria p_Acidobacteriota c_Acidobacteriae o_Acidobacteriales f_Koribacteraceae	91.2	2.7	63.6	3575390	349	0.05	0	1	21	14
Bin.002_Pasture	Pasture	d_Bacteria p_Actinobacteriota c_Acidimicrobiia o_IMCC26256	97.4	2.1	69.5	4392436	177	0.14	1	1	48	20
Bin.005_Pasture	Pasture	d_Bacteria p_Eremiobacterota c_Eremiobacteria o_Baltobacterales f_Baltobacteraceae g_Cybelea	93.5	0.9	62.3	2468445	164	0.07	1	1	32	15
Bin.006_Pasture	Pasture	d_Bacteria p_Desulfobacterota_B c_Binatia o_Binatales f_Binataceae	90.7	6.0	57.4	4826547	379	0.07	0	1	34	18
Bin.020_Pasture	Pasture	d_Bacteria p_Dormibacterota c_Dormibacteria o_Dormibacterales f_Dormibacteraceae g_40 CM-4-65-16	100.0	0.9	67.1	3021951	46	0.10	1	0	71	20
Bin.027_Pasture	Pasture	d_Bacteria p_Proteobacteria c_Alphaproteobacteria o_Rhizobiales f_Hyphomicrobiaceae g_AWTP1-13	93.5	4.4	64.4	4444876	388	0.10	1	0	21	13
Bin.031_Pasture	Pasture	d_Bacteria p_Proteobacteria c_Alphaproteobacteria o_Acetobacterales f_Acetobacteraceae g_Palsa-883	99.3	2.2	63.2	6420847	156	0.26	1	0	45	19
Bin.034_Pasture	Pasture	d_Archaea p_Thermoproteota c_Nitrososphaeria o_Nitrososphaerales f_Nitrososphaeraceae g_UBA10452 s_UBA10452 sp009898475	95.2	1.0	40.5	1180365	33	0.03	1	0	39	18
Bin.035_Pasture	Pasture	d_Archaea p_Halobacteriota c_Methanosarcinia o_Methanosarcinales f_Methanosarcinaceae g_Methanosarcina	78.4	0.7	39.9	2614668	335	0.03	1	0	44	18

MAGs selected based on their completeness (>90 %) and contamination (<5 %) scores. Bin.006_Pasture and Bin.035_Pasture were also included due to their environmental relevance.

Despite their near-completeness (>90 % of completeness and <5 % of contamination), our selected bins did not meet all MIMAG standards for high-quality drafts due to the absence of certain rRNA genes or tRNAs [35] (Table 1). However, four MAGs possessed the 16S rRNA gene; nine, the 5S rRNA gene; and nine, tRNAs for at least 18 amino acids. No sequences of the 23S rRNA gene could be found in the MAGs.

### Functional characterization of MAGs and biogeochemical relevance

**Fig. 3.**
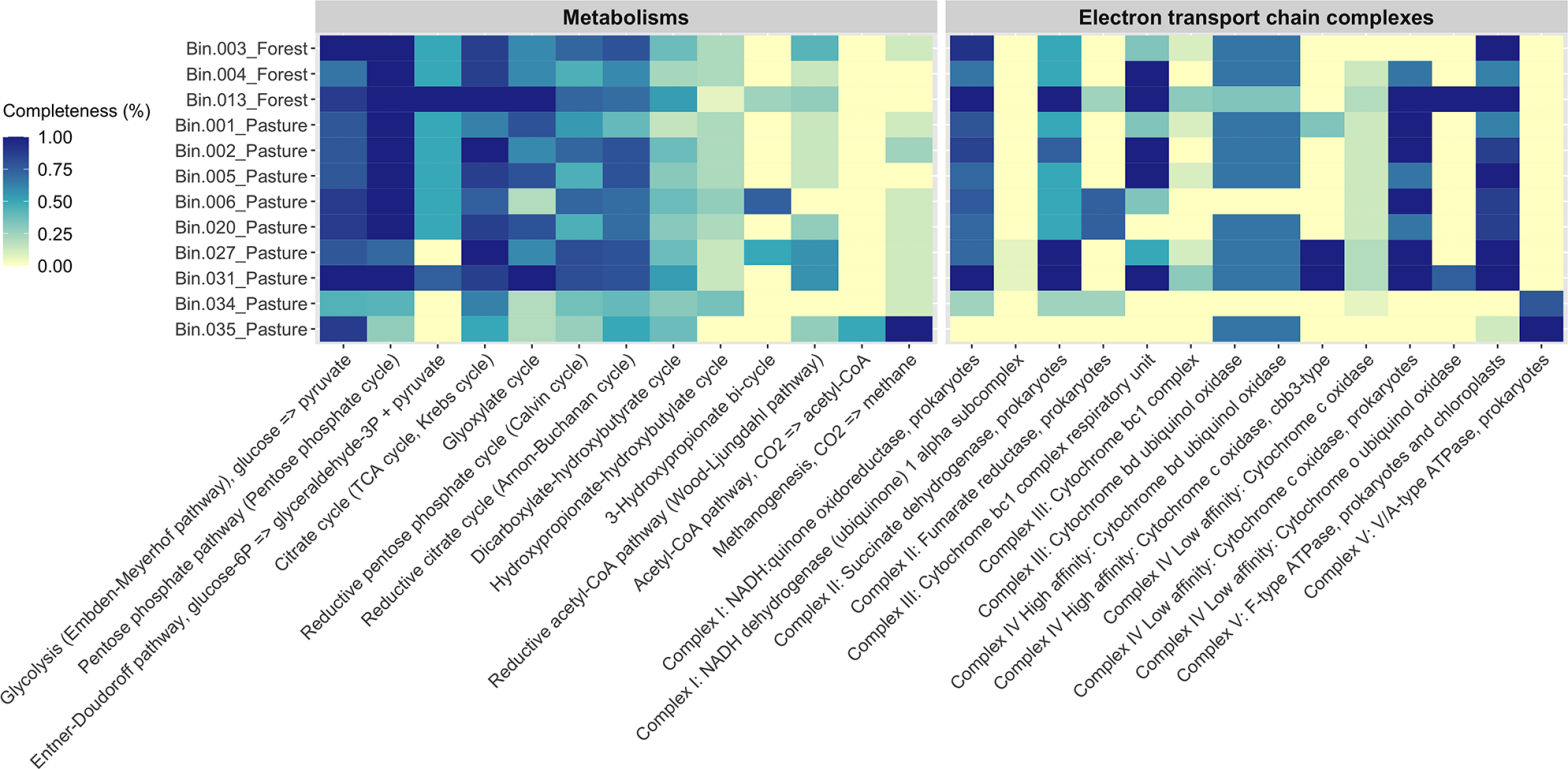
DRAM annotations of the selected forest and pasture metagenome-assembled genomes (MAGs). The MAGs were selected based on their completeness (>90 %) and contamination (<5 %) scores. Bin.006_Pasture and Bin.035_Pasture were also included due to their environmental relevance. The colours in the heatmap represent the completeness of relevant pathways and electron transport chain complexes in each MAG. The heatmap was created based on the DRAM output figure and only depicts modules that were present in at least one MAG. The list of genes associated with each module is available at https://www.doi.org/10.25982/91640.61/1870603.

Genes related to glycolysis (Embden-Meyerhof pathway), pentose phosphate pathway (pentose phosphate cycle), citrate (TCA or Krebs cycle), glyoxylate, reductive pentose phosphate (Calvin cycle), reductive citrate (Arnon-Buchanan cycle), and dicarboxylate-hydroxybutyrate cycles were found in all selected MAGs (Fig. 3). Some of them featured the full pathways for relevant metabolisms, including glycolysis, pentose phosphate, and Entner-Doudoroff pathways – this alternative pathway to glycolysis is most common in Gram-negative bacteria [43], and it was detected in the proteobacterial MAG from the family *Burkholderiaceae* (Bin.013_Forest, genus *Paraburkholderia *) – as well as citrate and glyoxylate cycles. The latter allows organisms to grow on acetate or fatty acids as sole carbon sources [44]. Several electron transport chain complexes (I - V), associated with aerobic respiration, have also been found to be fully covered in the MAGs. No complete carbon fixation pathways were annotated in our genomes.

Previous studies have reported a CH_4_ sink-to-source shift after forest-to-pasture conversion in the Amazon [13, 15, 45–48], also revealing that pasture soils usually harbour a higher abundance of CH_4_-producing archaea (methanogens) than forest soils [13–15]. Furthermore, using our microcosm experiment dataset, we demonstrated that increased soil moisture intensified soil CH_4_ emissions and related microbial responses driven by forest-to-pasture conversion [15]. In consequence, here, we were able to recover a MAG of the genus *Methanosarcina* from the pasture (Bin.035_Pasture), a group of strictly anaerobic CH_4_-producing archaea [36], which was much more abundant in our pasture soils than in the forest (Fig. 2). As expected, the methanogenesis pathway was fully detected in this novel genome.

**Fig. 4.**
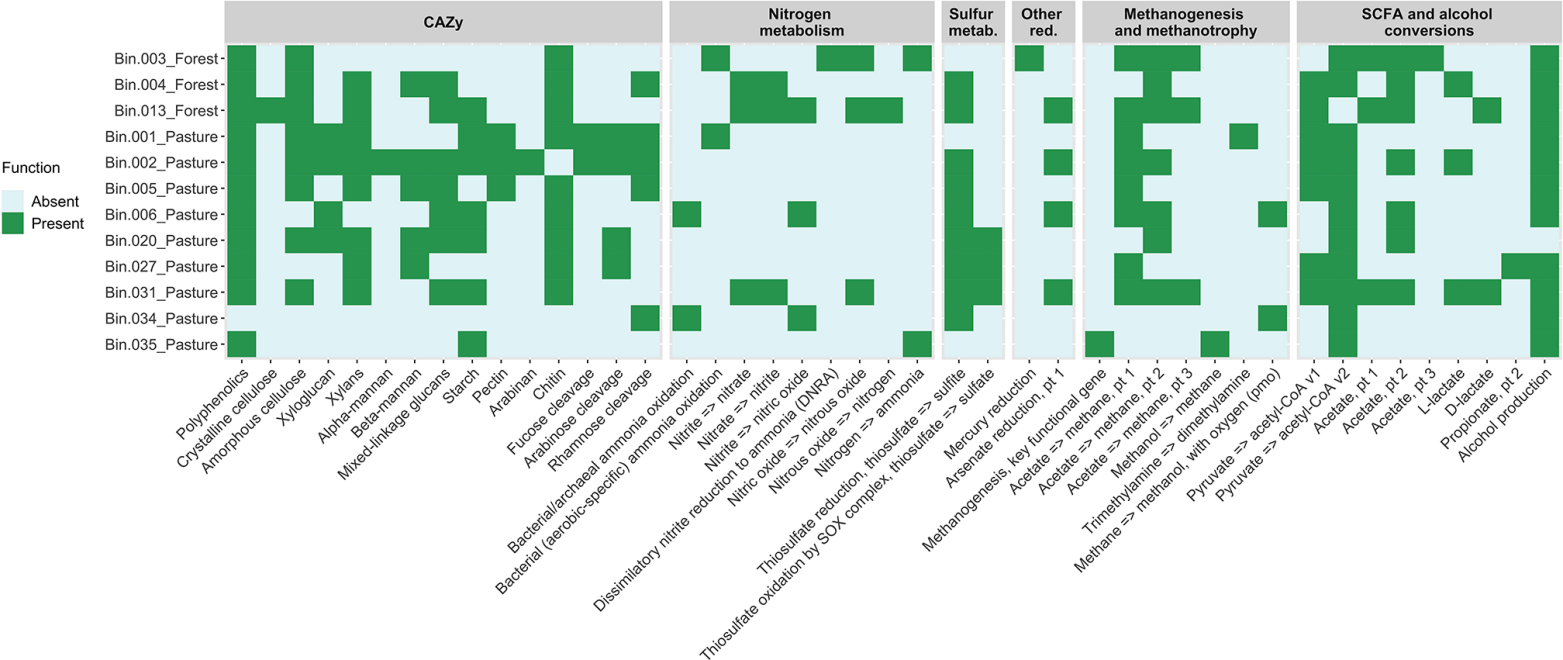
DRAM annotations of the selected forest and pasture metagenome-assembled genomes (MAGs). The MAGs were selected based on their completeness (>90 %) and contamination (<5 %) scores. Bin.006_Pasture and Bin.035_Pasture were also included due to their environmental relevance. The colours in the heatmap represent the presence or absence of relevant metabolic functions in each MAG. The heatmap was created based on the DRAM output figure and only depicts modules that were present in at least one MAG. The list of genes associated with each module is available at https://www.doi.org/10.25982/91640.61/1870603. CAZy, carbohydrate-active enzymes; metab., metabolism; red., reductases; SCFA, short-chain fatty acids.

The degradation of organic molecules by soil microbial communities is a crucial step in the carbon cycle [49]. We further investigated the presence of carbohydrate-active enzymes (CAZymes) genes in the MAGs, revealing genes related to the cleavage of polyphenolics and complex carbohydrates, such as chitin, amorphous cellulose, xylans, mixed-linkage glucans, and starch (Fig. 4). The pasture MAGs from the classes *Acidimicrobiia* (Bin.002_Pasture), *Acidobacteriae* (Bin.001_Pasture), *Dormibacteria* (Bin.020_Pasture), and *Eremiobacteria* (Bin.005_Pasture) were found to have the potential to degrade the highest number of substrates. In fact, Silva-Olaya *et al*. [50] suggested a higher mineralization potential by the soil microbiota in pastures compared to forest soils of the Colombian Amazon. Understanding the different microbial strategies to convert biomass in Amazonian soils is essential to unveil their potential ecosystem services in these environments.

Regarding other fundamental biogeochemical cycles, sulphur is considered one of the most important elements for life, and its related microbial oxidation and reduction processes occur in several ecosystems [51]. Genes associated with the thiosulphate reduction to sulphite (*rdlA* gene) [52] and thiosulphate oxidation to sulphate through the sulphur oxidation (Sox) enzyme system (*soxXYZABCD* genes) [53, 54] have also been found in some MAGs (Fig. 4). The alphaproteobacterial MAGs of the *Hyphomicrobiaceae* and *Acetobacteraceae* families possess five and six Sox genes, respectively (*soxA*, *soxB*, *soxX*, *soxY*, and *soxZ* in Bin.027_Pasture; and *soxA*, *soxC*, *soxD*, *soxX*, *soxY*, and *soxZ* in Bin.031_Pasture).

Genes related to nitrogen-transforming processes – such as nitrogen fixation, nitrification, denitrification, and dissimilatory nitrate reduction to ammonium (DNRA) – could also be detected in the MAGs, including *nifD*, *nifH*, and *nifK* for nitrogenase in Bin.003_Forest and Bin.035_Pasture; *narK*/*nrtP*/*nasA* for nitrate/nitrite transporter, *narG*/*narZ*/*nxrA* and *narH*/*narY*/*nxrB* for nitrate reductase/nitrite oxidoreductase, and *narI*/*narV* for nitrate reductase in Bin.004_Forest, Bin.013_Forest, and Bin.031_Pasture; *nasA* for assimilatory nitrate reductase in Bin.013_Forest; *nrfA* and *nrfH* for cytochrome c nitrite reductase in Bin.003_Forest; *nirB* and *nirD* for nitrite reductase (NADH) in Bin.013_Forest; *hao* for hydroxylamine dehydrogenase (HAO) in Bin.003_Forest and Bin.001_Pasture; and *nirK* for nitrite reductase (NO-forming) in Bin.013_Forest, Bin.006_Pasture, and Bin.034_Pasture. It is worth mentioning that the *nirK* gene of Bin.034_pasture may be related to nitrification, as it has been reported that this gene may oxidize hydroxylamine to N_2_O in ammonia-oxidizing archaea, functioning as a bacteria-like HAO [55, 56].

The nitric oxide reductase, responsible for the microbial reduction of nitric oxide (NO) to N_2_O – the main source of this greenhouse gas [57], was detected in three bacterial MAGs from the families *Geobacteraceae* (Bin.003_Forest with *norB*), *Burkholderiaceae* (Bin.013_Forest with *norB* and *norC*), and *Acetobacteraceae* (Bin.031_Pasture with *norB*). On the other hand, a nitrous oxide reductase (*nosZ*) that reduces N_2_O to dinitrogen [4] is encoded by the *Burkholderiaceae* MAG. In previous studies in the Amazon region, this important gene for N_2_O consumption was found in higher abundance in forests in comparison with pasture soils [48, 58].

Methane/ammonia monooxygenase genes were detected in our archaea from the *Nitrososphaeraceae* family (Bin.034_Pasture with *pmoA-amoA* and *pmoB-amoB*). Members of this family are capable of oxidizing ammonia, with a few soil isolates [59–63]. The pasture MAG from the class *Binatia* (Bin.006_Pasture, order *Binatales *) also contains *pmoA-amoA*, *pmoB-amoB*, and *pmoC-amoC. Binatota* is a yet-uncultured, poorly characterized candidate phylum, but some of its members have been recently suggested to be involved in CH_4_ oxidation [37]. This recent study revealed that 11 MAGs – of the orders *Bin18* and *Binatales* – from a total of 108 encode copper membrane monooxygenases (CuMMOs), an enzyme family that includes the particulate methane monooxygenase (pMMO) [37]. Therefore, these microorganisms, not yet considered in studies on the Amazonian soil CH_4_ cycle but more abundant in our pasture soils, may potentially be related to the consumption of this greenhouse gas.

Still considering the CH_4_ cycle, as expected, the pasture *Methanosarcina* MAG (Bin.035_Pasture) possesses the genes (*mcrA*, *mcrB*, and *mcrG*) for the methyl-coenzyme M reductase (MCR), the terminal enzyme of the methanogenesis process that catalyses the formation of CH_4_ from methyl-coenzyme M [64]. The genus *Methanosarcina* is composed of versatile organisms that can produce CH_4_ through the main methanogenic pathways (hydrogenotrophic, acetoclastic, and/or methylotrophic) [36] and, in addition to other methanogenesis-related genes, this genome codes for heterodisulfide reductases (Hdr, genes *hdrB1*, *hdrB2*, *hdrC1*, *hdrC2*, *hdrD*, and *hdrE*). It also carries *mtaA*, *mtaB*, and *mtaC* genes (for [methyl-Co(III) methanol/glycine betaine-specific corrinoid protein]:coenzyme M methyltransferase, methanol--−5-hydroxybenzimidazolylcobamide Co-methyltransferase, and methanol corrinoid protein, respectively) and *mtbC* (for dimethylamine corrinoid protein), considered molecular markers for methylotrophic methanogenic archaea [65]. Lastly, the genome from the *Koribacteraceae* family of *Acidobacteriota* (Bin.001_Pasture) possesses the *mttB* gene (for trimethylamine---corrinoid protein Co-methyltransferase).

Amazonian soils are naturally rich in mercury [66] and, along with numerous other relevant functions observed in the genomes (Fig. 4), the forest MAG from the *Geobacteraceae* family of *Desulfobacterota* _F (Bin.003_Forest) also encodes a mercuric reductase, related to mercury detoxification, an important feature for the bioremediation of contaminated environments [67]. Furthermore, genes associated with acetate metabolism – a short-chain fatty acid used as an energy and carbon source for several microorganisms [68], including certain *Methanosarcina* species [36] – were also present in the MAGs (most notably, *acs* for acetyl-CoA synthetase, *ackA* for acetate kinase, and *pta* for phosphate acetyltransferase in Bin.003_Forest, Bin.013_Forest, and Bin.031_Pasture, and *ACH1* for acetyl-CoA hydrolase in Bin.003_Forest).

## Conclusion

In conclusion, genome-resolved metagenomics revealed potentially novel genomes from forest and pasture soils of the Eastern Amazon. This approach can expand our knowledge about the microbial communities from Amazonian soils and reveal the functional potential of novel or underrepresented microbes, thus helping us to understand their ecological roles in this environment. Considering the relationship of these genomes with critical and closely linked biogeochemical cycles, our results also constitute an important resource for further studies on the functional responses of Amazonian soil microbial communities in light of land-use and climate change.

## Supplementary Data

Supplementary material 1

## References

[1] Verstraete W, Mertens B, Doelman P, Eijsackers HJP (2004). Developments in Soil Science.

[2] Fierer N, Wood SA, Bueno de Mesquita CP (2021). How microbes can, and cannot, be used to assess soil health. Soil Biol Biochem.

[3] Jansson JK, Hofmockel KS (2020). Soil microbiomes and climate change. Nat Rev Microbiol.

[4] Stein LY (2020). The long-term relationship between microbial metabolism and greenhouse gases. Trends Microbiol.

[5] Lehmann J, Bossio DA, Kögel-Knabner I, Rillig MC (2020). The concept and future prospects of soil health. Nat Rev Earth Environ.

[6] Cameron EK, Martins IS, Lavelle P, Mathieu J, Tedersoo L (2018). Global gaps in soil biodiversity data. Nat Ecol Evol.

[7] Guerra CA, Heintz-Buschart A, Sikorski J, Chatzinotas A, Guerrero-Ramírez N (2020). Blind spots in global soil biodiversity and ecosystem function research. Nat Commun.

[8] Heckenberger MJ, Russell JC, Toney JR, Schmidt MJ (2007). The legacy of cultural landscapes in the Brazilian Amazon: implications for biodiversity. Philos Trans R Soc Lond B Biol Sci.

[9] (2021). Instituto Nacional de Pesquisas Espaciais - INPE. TerraBrasilis.

[10] Rodrigues JLM, Pellizari VH, Mueller R, Baek K, Jesus EC (2013). Conversion of the Amazon rainforest to agriculture results in biotic homogenization of soil bacterial communities. Proc Natl Acad Sci U S A.

[11] Paula FS, Rodrigues JLM, Zhou J, Wu L, Mueller RC (2014). Land use change alters functional gene diversity, composition and abundance in Amazon forest soil microbial communities. Mol Ecol.

[12] Mendes LW, de Lima Brossi MJ, Kuramae EE, Tsai SM (2015). Land-use system shapes soil bacterial communities in Southeastern Amazon region. Appl Soil Ecol.

[13] Meyer KM, Morris AH, Webster K, Klein AM, Kroeger ME (2020). Belowground changes to community structure alter methane-cycling dynamics in Amazonia. Environ Int.

[14] Kroeger ME, Meredith LK, Meyer KM, Webster KD, de Camargo PB (2021). Rainforest-to-pasture conversion stimulates soil methanogenesis across the Brazilian Amazon. ISME J.

[15] Venturini AM, Dias NMS, Gontijo JB, Yoshiura CA, Paula FS (2022). Increased soil moisture intensifies the impacts of forest-to-pasture conversion on methane emissions and methane-cycling communities in the Eastern Amazon. Environ Res.

[16] Saheb Kashaf S, Almeida A, Segre JA, Finn RD (2021). Recovering prokaryotic genomes from host-associated, short-read shotgun metagenomic sequencing data. Nat Protoc.

[17] Parks DH, Rinke C, Chuvochina M, Chaumeil P-A, Woodcroft BJ (2017). Recovery of nearly 8,000 metagenome-assembled genomes substantially expands the tree of life. Nat Microbiol.

[18] Pasolli E, Asnicar F, Manara S, Zolfo M, Karcher N (2019). Extensive unexplored human microbiome diversity revealed by over 150,000 genomes from metagenomes spanning age, geography, and lifestyle. Cell.

[19] Nayfach S, Roux S, Seshadri R, Udwary D, Varghese N (2021). A genomic catalog of Earth’s microbiomes. Nat Biotechnol.

[20] Kroeger ME, Delmont TO, Eren AM, Meyer KM, Guo J (2018). New biological insights into how deforestation in Amazonia affects soil microbial communities using metagenomics and metagenome-assembled genomes. Front Microbiol.

[21] Lemos LN, Manoharan L, Mendes LW, Venturini AM, Pylro VS (2020). Metagenome assembled-genomes reveal similar functional profiles of CPR/patescibacteria phyla in soils. Environ Microbiol Rep.

[22] Venturini AM, Nakamura FM, Gontijo JB, da França AG, Yoshiura CA (2020). Robust DNA protocols for tropical soils. Heliyon.

[23] Arkin AP, Cottingham RW, Henry CS, Harris NL, Stevens RL (2018). KBase: The United States Department of Energy Systems Biology Knowledgebase. Nat Biotechnol.

[24] Andrews S (2010). FastQC: A quality control tool for high throughput sequence data. http://www.bioinformatics.babraham.ac.uk/projects/fastqc.

[25] Ewels P, Magnusson M, Lundin S, Käller M (2016). MultiQC: summarize analysis results for multiple tools and samples in a single report. Bioinformatics.

[26] Bolger AM, Lohse M, Usadel B (2014). Trimmomatic: a flexible trimmer for Illumina sequence data. Bioinformatics.

[27] Li D, Liu C-M, Luo R, Sadakane K, Lam T-W (2015). MEGAHIT: an ultra-fast single-node solution for large and complex metagenomics assembly via succinct de Bruijn graph. Bioinformatics.

[28] Kang DD, Froula J, Egan R, Wang Z (2015). MetaBAT, an efficient tool for accurately reconstructing single genomes from complex microbial communities. PeerJ.

[29] Wu Y-W, Simmons BA, Singer SW (2016). MaxBin 2.0: an automated binning algorithm to recover genomes from multiple metagenomic datasets. Bioinformatics.

[30] Alneberg J, Bjarnason BS, de Bruijn I, Schirmer M, Quick J (2014). Binning metagenomic contigs by coverage and composition. Nat Methods.

[31] Sieber CMK, Probst AJ, Sharrar A, Thomas BC, Hess M (2018). Recovery of genomes from metagenomes via a dereplication, aggregation and scoring strategy. Nat Microbiol.

[32] Parks DH, Imelfort M, Skennerton CT, Hugenholtz P, Tyson GW (2015). CheckM: assessing the quality of microbial genomes recovered from isolates, single cells, and metagenomes. Genome Res.

[33] Chaumeil P-A, Mussig AJ, Hugenholtz P, Parks DH (2019). GTDB-Tk: a toolkit to classify genomes with the Genome Taxonomy Database. Bioinformatics.

[34] Jain C, Rodriguez-R LM, Phillippy AM, Konstantinidis KT, Aluru S (2018). High throughput ANI analysis of 90K prokaryotic genomes reveals clear species boundaries. Nat Commun.

[35] Bowers RM, Kyrpides NC, Stepanauskas R, Harmon-Smith M, Doud D (2017). Minimum information about a single amplified genome (MISAG) and a metagenome-assembled genome (MIMAG) of bacteria and archaea. Nat Biotechnol.

[36] Wagner D, Trujillo ME, Dedysh S, DeVos P, Hedlund B, Kämpfer P (2020). Bergey’s Manual of Systematics of Archaea and Bacteria.

[37] Murphy CL, Sheremet A, Dunfield PF, Spear JR, Stepanauskas R (2021). Genomic analysis of the yet-uncultured Binatota reveals broad methylotrophic, alkane-degradation, and pigment production capacities. mBio.

[38] Langmead B, Salzberg SL (2012). Fast gapped-read alignment with Bowtie 2. Nat Methods.

[39] Shaffer M, Borton MA, McGivern BB, Zayed AA, La Rosa SL (2020). DRAM for distilling microbial metabolism to automate the curation of microbiome function. Nucleic Acids Res.

[40] Wickham H (2016). Ggplot2: Elegant Graphics for Data Analysis.

[41] Brunson JC, Read QD (2020). ggalluvial: Alluvial plots in “ggplot2”. R package version 0.12.3. http://corybrunson.github.io/ggalluvial/.

[42] R Core Team (2020). R: A language and environment for statistical computing. R Foundation for statistical computing. https://www.R-project.org/.

[43] Conway T (1992). The Entner-Doudoroff pathway: history, physiology and molecular biology. FEMS Microbiol Rev.

[44] Kornberg HL, Krebs HA (1957). Synthesis of cell constituents from C_2_-units by a modified tricarboxylic acid cycle. Nature.

[45] Steudler PA, Melillo JM, Feigl BJ, Neill C, Piccolo MC (1996). Consequence of forest-to-pasture conversion on CH_4_ fluxes in the Brazilian Amazon Basin. J Geophys Res.

[46] Verchot LV, Davidson EA, Cattânio JH, Ackerman IL (2000). Land-use change and biogeochemical controls of methane fluxes in soils of Eastern Amazonia. Ecosystems.

[47] Fernandes SAP, Bernoux M, Cerri CC, Feigl BJ, Piccolo MC (2002). Seasonal variation of soil chemical properties and CO_2_ and CH_4_ fluxes in unfertilized and P-fertilized pastures in an Ultisol of the Brazilian Amazon. Geoderma.

[48] Lammel DR, Feigl BJ, Cerri CC, Nüsslein K (2015). Specific microbial gene abundances and soil parameters contribute to C, N, and greenhouse gas process rates after land use change in Southern Amazonian Soils. Front Microbiol.

[49] Andrade AC, Fróes A, Lopes FÁC, Thompson FL, Krüger RH (2017). Diversity of microbial carbohydrate-active enZYmes (CAZYmes) associated with freshwater and soil samples from Caatinga biome. Microb Ecol.

[50] Silva-Olaya AM, Mora-Motta DA, Cherubin MR, Grados D, Somenahally A (2021). Soil enzyme responses to land use change in the tropical rainforest of the Colombian Amazon region. PLoS ONE.

[51] Kumar U, Panneerselvam P, Gupta V, Manjunath M, Priyadarshinee P, Adhya TK, Lal B, Mohapatra B, Paul D, Das S (2018). Advances in Soil Microbiology: Recent Trends and Future Prospects. Microorganisms for Sustainability.

[52] Ravot G, Casalot L, Ollivier B, Loison G, Magot M (2005). *rdlA*, a new gene encoding a rhodanese-like protein in *Halanaerobium congolense* and other thiosulfate-reducing anaerobes. Res Microbiol.

[53] Friedrich CG, Bardischewsky F, Rother D, Quentmeier A, Fischer J (2005). Prokaryotic sulfur oxidation. Curr Opin Microbiol.

[54] Rother D, Orawski G, Bardischewsky F, Friedrich CG (2005). SoxRS-mediated regulation of chemotrophic sulfur oxidation in *Paracoccus pantotrophus*. Microbiology (Reading).

[55] Kobayashi S, Hira D, Yoshida K, Toyofuku M, Shida Y (2018). Nitric oxide production from nitrite reduction and hydroxylamine oxidation by copper-containing dissimilatory nitrite reductase (nirk) from the aerobic ammonia-oxidizing archaeon, *Nitrososphaera viennensis*. Microbes Environ.

[56] Wu L, Chen X, Wei W, Liu Y, Wang D (2020). A critical review on nitrous oxide production by ammonia-oxidizing archaea. Environ Sci Technol.

[57] Kuypers MMM, Marchant HK, Kartal B (2018). The microbial nitrogen-cycling network. Nat Rev Microbiol.

[58] Lammel DR, Nüsslein K, Tsai SM, Cerri CC (2015). Land use, soil and litter chemistry drive bacterial community structures in samples of the rainforest and Cerrado (Brazilian Savannah) biomes in Southern Amazonia. Eur J Soil Biol.

[59] Kim J-G, Jung M-Y, Park S-J, Rijpstra WIC, Sinninghe Damsté JS (2012). Cultivation of a highly enriched ammonia-oxidizing archaeon of thaumarchaeotal group I.1b from an agricultural soil. Environ Microbiol.

[60] Stieglmeier M, Klingl A, Alves RJE, Rittmann SK-MR, Melcher M (2014). *Nitrososphaera viennensis* gen. nov., sp. nov., an aerobic and mesophilic, ammonia-oxidizing archaeon from soil and a member of the archaeal phylum *Thaumarchaeota*. Int J Syst Evol Microbiol.

[61] Zhalnina KV, Dias R, Leonard MT, Dorr de Quadros P, Camargo FAO (2014). Genome sequence of Candidatus *Nitrososphaera evergladensis* from group I.1b enriched from Everglades soil reveals novel genomic features of the ammonia-oxidizing archaea. PLoS ONE.

[62] Kerou M, Schleper C, Trujillo ME, Dedysh S, DeVos P, Hedlund B, Kämpfer P (2016). Bergey’s Manual of Systematics of Archaea and Bacteria.

[63] Lehtovirta-Morley LE, Ross J, Hink L, Weber EB, Gubry-Rangin C (2016). Isolation of “*Candidatus* Nitrosocosmicus franklandus”, a novel ureolytic soil archaeal ammonia oxidiser with tolerance to high ammonia concentration. FEMS Microbiol Ecol.

[64] Luton PE, Wayne JM, Sharp RJ, Riley PW (2002). The *mcrA* gene as an alternative to 16S rRNA in the phylogenetic analysis of methanogen populations in landfill. Microbiology (Reading).

[65] Dziewit L, Pyzik A, Romaniuk K, Sobczak A, Szczesny P (2015). Novel molecular markers for the detection of methanogens and phylogenetic analyses of methanogenic communities. Front Microbiol.

[66] Siqueira GW, Aprile F, Irion G, Braga ES (2018). Mercury in the Amazon basin: human influence or natural geological pattern?. J South Am Earth Sci.

[67] Barkay T, Miller SM, Summers AO (2003). Bacterial mercury resistance from atoms to ecosystems. FEMS Microbiol Rev.

[68] Zhuang G-C, Peña-Montenegro TD, Montgomery A, Montoya JP, Joye SB (2019). Significance of acetate as a microbial carbon and energy source in the water column of Gulf of Mexico: implications for marine carbon cycling. Global Biogeochem Cycles.

