# Supplementary material for: Genome-resolved metagenomics reveals novel archaeal and bacterial genomes from Amazonian forest and pasture soils
Source: Microb Genom. 2022 Jul 27;8(7):mgen000853. doi: 10.1099/mgen.0.000853 (PMC9455692; doi:10.1099/mgen.0.000853)
Supplement: Supplementary material 1 [file mgen-8-853-s001.pdf]

Supporting information

Results

Supplementary Table S1. Detailed information (taxonomic classification, completeness, contamination, quality score, GC content, genome size, number of contigs, longest contig, N50, number of ambiguous bases and predicted genes) of all forest and pasture metagenome-assembled genomes (MAGs).

**MAG**	**Site**	**GTDB classification**	**Completeness (%)**	**Contamination (%)**	**Quality score**	**GC content (%)**	**Genome size (bp)**	**No. of contigs**	**Longest contig (bp)**	**N50 (bp)**	**No. of ambiguous bases**	**No. of predicted genes**
Bin.002_Forest	Forest	d_Bacteria p_Actinobacteriota c_Thermoleophilia o_Solirubrobacterales f_Solirubrobacteraceae g_Palsa-744	82.0	8.1	41.4	71.2	2,682,473	279	95,740	13,117	0	2,777
Bin.003_Forest	Forest	d_Bacteria p_Desulfobacterota_F c_Desulfuromonadia o_Geobacterales f_Geobacteraceae	95.7	1.9	86.0	59.0	4,438,015	103	203,831	68,187	0	4,087
Bin.004_Forest	Forest	d_Bacteria p_Eremiobacterota c_Eremiobacteria o_Baltobacterales f_Baltobacteraceae g_Aquilonibacter	93.8	3.5	76.3	59.3	3,739,922	442	91,620	12,707	0	4,049
Bin.005_Forest	Forest	d_Bacteria p_Dormibacterota c_Dormibacteria o_Dormibacterales f_Dormibacteraceae g_Dormibacter	79.2	2.8	65.3	69.5	2,809,910	293	53,542	11,006	0	2,996

Bin.006_Forest	Forest	d_Bacteria<br>p_Actinobacteriota<br>c_Thermoleophilia<br>o_Solirubrobacterales<br>f_Solirubrobacteraceae<br>g_Palsa-744	74.2	3.6	56.4	68.6	2,340,103	313	33,251	10,316	0	2,491
Bin.008_Forest	Forest	d_Bacteria<br>p_Acidobacteriota<br>c_Acidobacteriae<br>o_Acidobacteriales<br>f_Gp1-AA117<br>g_Gp1-AA17	75.0	9.4	28.0	58.7	5,178,656	808	42,915	7,423	0	5,023
Bin.009_Forest	Forest	d_Bacteria<br>p_Proteobacteria<br>c_Alphaproteobacteria<br>o_ATCC43930<br>f_Stellaceae<br>g_AP-15	56.6	3.5	39.2	62.6	4,601,747	1,063	31,408	4,289	0	5,133
Bin.010_Forest	Forest	d_Bacteria<br>p_Desulfobacterota_F<br>c_Desulfuromonadia<br>o_Geobacterales<br>f_Geobacteraceae<br>g_Geomonas	63.9	4.2	42.8	64.3	2,940,354	662	28,921	4,430	0	3,053
Bin.011_Forest	Forest	d_Bacteria<br>p_Actinobacteriota<br>c_Actinomycetia<br>o_Mycobacteriales<br>f_Mycobacteriaceae<br>g_Mycobacterium	73.7	7.7	35.0	65.6	3,803,225	768	20,344	5,387	0	4,227
Bin.013_Forest	Forest	d_Bacteria<br>p_Proteobacteria<br>c_Gammaproteobacteria<br>o_Burkholderiales<br>f_Burkholderiaceae<br>g_Paraburkholderia<br>s_Paraburkholderia<br>sp004298475	95.7	3.8	76.9	63.5	5,591,535	78	374,273	141,664	0	5,109

Bin.016_Forest	Forest	d_Bacteria<br>p_Acidobacteriota<br>c_Acidobacteriae<br>o_Acidobacteriales<br>f_Koribacteraceae<br>g_Gp1-AA122	69.3	0.4	67.2	57.4	2,401,684	197	45,383	17,376	0	2,211
Bin.001_Pasture	Pasture	d_Bacteria<br>p_Acidobacteriota<br>c_Acidobacteriae<br>o_Acidobacteriales<br>f_Koribacteraceae	91.2	2.7	77.8	63.6	3,575,390	349	61,427	13,744	0	3,401
Bin.002_Pasture	Pasture	d_Bacteria<br>p_Actinobacteriota<br>c_Acidimicrobiia<br>o_IMCC26256	97.4	2.1	86.7	69.5	4,392,436	177	128,742	41,908	0	4,401
Bin.004_Pasture	Pasture	d_Bacteria<br>p_Actinobacteriota<br>c_Acidimicrobiia<br>o_Acidimicrobiales<br>f_QHCF01<br>g_AC-9	95.4	7.1	59.8	68.7	4,368,694	304	71,218	21,897	0	4,466
Bin.005_Pasture	Pasture	d_Bacteria<br>p_Eremiobacterota<br>c_Eremiobacteria<br>o_Baltobacterales<br>f_Baltobacteraceae<br>g_Cybelea	93.5	0.9	88.8	62.3	2,468,445	164	72,579	24,142	0	2,550
Bin.006_Pasture	Pasture	d_Bacteria<br>p_Desulfobacterota_B<br>c_Binatia<br>o_Binatales<br>f_Binataceae	90.7	6.0	60.8	57.4	4,826,547	379	69,352	17,712	0	4,740

Bin.007_Pasture	Pasture	d_Bacteria<br>p_Verrucomicrobiota<br>c_Verrucomicrobiae<br>o_Chthoniobacterales<br>f_UBA10450<br>g_Udaeobacter	75.5	5.5	48.2	53.8	2,561,871	559	18,677	4,716	0	2,754
Bin.008_Pasture	Pasture	d_Bacteria<br>p_Proteobacteria<br>c_Alphaproteobacteria<br>o_Rhizobiales<br>f_Rhodomicrobiaceae<br>g_Rhodomicrobium	50.0	1.9	40.6	58.9	2,394,149	504	21,688	4,999	0	2,650
Bin.009_Pasture	Pasture	d_Bacteria<br>p_Actinobacteriota<br>c_Actinomycetia<br>o_Mycobacteriales<br>f_Pseudonocardiaceae	62.3	3.0	47.1	70.1	4,152,510	793	67,821	5,770	0	4,290
Bin.010_Pasture	Pasture	d_Bacteria<br>p_Proteobacteria<br>c_Alphaproteobacteria<br>o_Rhizobiales<br>f_Beijerinckiaceae	75.2	4.2	54.5	63.9	4,051,986	653	41,077	7,235	0	4,253
Bin.012_Pasture	Pasture	d_Bacteria<br>p_Acidobacteriota<br>c_Acidobacteriae<br>o_Acidobacteriales<br>f_Gp1-AA112	84.6	1.0	79.9	55.5	4,618,347	314	76,729	19,858	0	4,148
Bin.013_Pasture	Pasture	d_Bacteria<br>p_Patescibacteria<br>c_Paceibacteria<br>o_UBA6257<br>f_2-01-FULL-56-20	56.1	1.3	49.7	54.6	822,780	91	38,094	12,712	0	922

Bin.015_Pasture	Pasture	d_Bacteria<br>p_Acidobacteriota<br>c_Acidobacteriae<br>o_Acidobacteriales<br>f_Koribacteraceae<br>g_Sulfotelmatobacter	89.3	3.0	74.4	56.2	5,345,900	242	202,522	35,033	0	4,710
Bin.017_Pasture	Pasture	d_Bacteria<br>p_Proteobacteria<br>c_Alphaproteobacteria<br>o_Rhizobiales<br>f_Xanthobacteraceae<br>g_Bradyrhizobium	90.9	8.6	48.1	63.2	6,166,337	570	69,733	15,891	0	6,318
Bin.018_Pasture	Pasture	d_Bacteria<br>p_Planctomycetota<br>c_Planctomycetes<br>o_Gemmatales<br>f_Gemmataceae<br>g_Gemmata	90.7	7.0	55.6	69.4	8,659,106	422	96,741	31,496	0	7,160
Bin.019_Pasture	Pasture	d_Bacteria<br>p_Acidobacteriota<br>c_Acidobacteriae<br>o_Acidobacteriales<br>f_CAINCZ01	61.5	0.9	57.0	59.8	3,239,453	465	41,531	7,680	0	3,019
Bin.020_Pasture	Pasture	d_Bacteria<br>p_Dormibacterota<br>c_Dormibacteria<br>o_Dormibacterales<br>f_Dormibacteraceae<br>g_40CM-4-65-16	100.0	0.9	95.4	67.1	3,021,951	46	515,562	130,694	0	3,172
Bin.021_Pasture	Pasture	d_Bacteria<br>p_Actinobacteriota<br>c_Actinomycetia<br>o_Mycobacteriales<br>f_Mycobacteriaceae<br>g_Mycobacterium	87.6	3.3	71.1	67.3	4,450,449	616	118,062	8,405	0	4,575

Bin.022_Pasture	Pasture	d_Bacteria<br>p_Actinobacteriota<br>c_Acidimicrobiia<br>o_IMCC26256<br>f_PALSA-610	68.2	4.2	47.1	67.9	3,788,828	736	32,875	5,588	0	4,238
Bin.024_Pasture	Pasture	d_Bacteria<br>p_Chloroflexota<br>c_Anaerolineae<br>o_Anaerolineales<br>f_Anaerolineaceae<br>g_DSYU01	54.6	1.9	45.1	59.8	2,117,388	522	52,973	3,993	0	2,224
Bin.025_Pasture	Pasture	d_Bacteria<br>p_Chloroflexota<br>c_Ktedonobacteria<br>o_Ktedonobacterales<br>f_JACDGC01	71.5	1.0	66.6	58.5	4,615,358	959	16,321	5,118	0	4,814
Bin.026_Pasture	Pasture	d_Bacteria<br>p_Proteobacteria<br>c_Gammaproteobacteria<br>o_Steroidobacterales<br>f_Steroidobacteraceae<br>g_13-2-20CM-66-19	94.6	7.9	55.3	66.5	4,589,768	135	150,417	61,874	0	4,143
Bin.027_Pasture	Pasture	d_Bacteria<br>p_Proteobacteria<br>c_Alphaproteobacteria<br>o_Rhizobiales<br>f_Hyphomicrobiaceae<br>g_AWTP1-13	93.5	4.4	71.6	64.4	4,444,876	388	76,939	15,535	0	4,283
Bin.028_Pasture	Pasture	d_Bacteria<br>p_Actinobacteriota<br>c_Thermoleophilia<br>o_Solirubrobacterales<br>f_Solirubrobacteraceae<br>g_Palsa-465	79.0	5.6	51.2	71.2	3,309,592	516	41,070	7,774	0	3,607

Bin.029_Pasture	Pasture	d_Bacteria<br>p_Actinobacteriota<br>c_Actinomycetia<br>o_Mycobacteriales<br>f_Jatrophihabitantaceae<br>g_WQZC01	92.2	9.8	43.5	68.4	5,511,069	443	63,959	17,777	0	5,575
Bin.031_Pasture	Pasture	d_Bacteria<br>p_Proteobacteria<br>c_Alphaproteobacteria<br>o_Acetobacterales<br>f_Acetobacteraceae<br>g_Palsa-883	99.3	2.2	88.3	63.2	6,420,847	156	305,695	74,508	0	6,385
Bin.034_Pasture	Pasture	d_Archaea<br>p_Thermoproteota<br>c_Nitrososphaeria<br>o_Nitrososphaerales<br>f_Nitrososphaeraceae<br>g_UBA10452<br>s_UBA10452 sp009898475	95.2	1.0	90.3	40.5	1,180,365	33	117,280	73,782	0	1,302
Bin.035_Pasture	Pasture	d_Archaea<br>p_Halobacteriota<br>c_Methanosarcinia<br>o_Methanosarcinales<br>f_Methanosarcinaceae<br>g_Methanosarcina	78.4	0.7	75.2	39.9	2,614,668	335	60,370	8,975	0	2,529
Bin.036_Pasture	Pasture	d_Bacteria<br>p_Patescibacteria<br>c_Microgenomatia<br>o_UBA12405<br>f_UBA12405<br>g_WARW01<br>s_WARW01 sp013387215	79.2	0.9	74.9	34.1	1,104,129	57	68,615	34,050	0	1,193

Bin.038_Pasture	Pasture	d_Bacteria<br>p_Patescibacteria<br>c_Doudnabacteria<br>o_UBA920<br>f_UBA920<br>g_PALSA-1336<br>s_PALSA-1336 sp013387205	70.5	0.0	70.5	42.9	1,273,264	45	115,665	41,246	0	1,304
Bin.039_Pasture	Pasture	d_Bacteria<br>p_Planctomycetota<br>c_Planctomycetes<br>o_Pirellulales	63.2	4.4	41.3	59.9	3,946,630	922	18,010	4,409	0	3,812
